# Detection of Human Cytomegalovirus *lncRNA4.9* in Breast Tumor Tissue Using RNA In Situ Hybridization

**DOI:** 10.1369/00221554261474562

**Published:** 2026-08-19

**Authors:** Arthur Morley-Bunker, Georgia Turner, Brooke Beardsley, Helen Morrin, John Pearson, Logan C. Walker, Margaret Currie

**Affiliations:** Department of Pathology & Molecular Medicine, University of Otago, Christchurch, Christchurch, New Zealand; Department of Pathology & Molecular Medicine, University of Otago, Christchurch, Christchurch, New Zealand; Anatomical Pathology, Canterbury Health Laboratories, Te Whatu Ora–Health New Zealand Waitaha Canterbury, Christchurch, New Zealand; He Taonga Tapu Cancer Society Tissue Bank, Christchurch, Aotearoa New Zealand; Department of Medicine, University of Otago, Christchurch, Christchurch, New Zealand; Department of Pathology & Molecular Medicine, University of Otago, Christchurch, Christchurch, New Zealand; Department of Pathology & Molecular Medicine, University of Otago, Christchurch, Christchurch, New Zealand

**Keywords:** breast cancer, human cytomegalovirus (HCMV), long non-coding RNA4.9 (lncRNA4.9), macrophages, RNAscope, tumor microenvironment

## Abstract

Human cytomegalovirus (HCMV) has been linked to tumor progression in several cancers, but its role in breast cancer remains uncertain. This study investigated HCMV long non-coding RNA4.9 (*lncRNA4.9*) in three breast cancer subtypes (ER+/PR+, *n*=25; HER2-positive *n*=24; triple-negative, *n*=26) and normal breast tissue (*n*=5) using RNAscope. Serum HCMV IgG and IgM levels were measured, and immunohistochemistry was used to assess the pan-macrophage marker CD68 in breast tissue. HCMV IgG seropositivity was more common in ER+ patients, while IgM positivity was more frequent in HER2-positive and triple-negative cases. HCMV *lncRNA4.9* signals were detected in over half of tumor samples, localizing to invasive tumor cells, stromal cells, adipose tissue, and ductal carcinoma in situ (DCIS). Although detection rates did not differ significantly between breast cancer subtypes, triple-negative tumors exhibited mid- to high-level *lncRNA4.9* expression. Immunohistochemical analysis demonstrated CD68+ cells present in most tumors and were more abundant in HCMV *lncRNA4.9*-positive samples. These cells co-localized with *lncRNA4.9*-positive cells at invasive margins, while in associated foci of DCIS, CD68+ cells were present in surrounding stroma. This study provides the first demonstration of HCMV *lncRNA4.9* in breast tissue and supports HCMV’s potential role in the biology of a subset of breast cancers.

## Introduction

Worldwide, female breast cancer is the most frequently diagnosed cancer and leading cause of cancer death in women, with approximately 2.3 million new cases and 666,000 deaths in 2022.^1,2^ For comparison, in Aotearoa New Zealand (NZ), a country with a population of just over 5.3 million people, breast cancer is also the most commonly diagnosed cancer in women. It affects one in nine women over their lifetime, with 3300 women diagnosed and over 650 deaths per year.^3^ Although some important risk factors for breast cancer are recognized, such as age, reproductive hormones, and inherited susceptibility,^4–10^ epidemiological studies of breast cancer indicate important risk factors are yet to be identified.^10^

Worldwide, over 15% of cancers (about 2.2 million cancer cases) are attributed to infectious agents each year,^11–13^ with almost two thirds of these cancers estimated to be due to viruses.^12,13^ Human cytomegalovirus (HCMV) is a human beta herpes virus which, in common with all herpes viruses, causes lifelong latent infection.^14^ HCMV seropositivity ranges from 50% to 100% in adults, with seroprevalence increasing with age and tending to be higher in low-resourced countries and socioeconomically disadvantaged groups.^15,16^ HCMV can infect many cell types but exhibits particular tropism toward cells of the myeloid lineage, including monocytes, macrophages, and dendritic cells.^16,17^ In adults, the immune system generally maintains HCMV in a latent state and most infections are subclinical.^14^ However, HCMV may cause severe disease and mortality in immunosuppressed individuals.^18,19^

In 1997, Richardson proposed some breast cancers may be caused by late exposure to a common virus such as HCMV.^20^ HCMV has been identified as a contributing risk factor in other cancer types including colorectal cancer and neuroblastomas,^21,22^ and epidemiological and laboratory studies have identified mechanisms by which HCMV could drive carcinogenesis and progression in breast cancer (see for review Richardson et al.^23^ and Herbein^24^). A review of published studies demonstrated that the majority provided some evidence to support a causal association between HCMV and breast cancer. However, the associations were neither consistent nor strong.^25^

Recent studies by Herbein and colleagues have provided the first compelling evidence that HCMV may act as an oncogenic virus in breast cancer.^26^ Human mammary epithelial cells were infected with a clinical isolate of HCMV, and the HCMV-transformed human mammary epithelial cells were subsequently injected into the mammary fat pads of immune-deficient mice.^26^ Biopsies revealed the tumors that developed in these mice were triple receptor negative [estrogen receptor (ER)−/progesterone receptor (PR)−/human epidermal growth factor receptor (HER2)−].^26^ Importantly, the authors detected the HCMV gene product long non-coding RNA4.9 (*lncRNA4.9*) in transformed human breast epithelial cells, mouse xenograft tumors, and biopsies from human triple-negative breast cancers (TNBC).^26,27^ *lncRNA4.9* is a regulator of HCMV DNA replication^28^ and is highly expressed by HCMV in both lytic (active) and latent (dormant) infection, making it a prime target for detection of HCMV.^29^

RNAscope is an RNA in situ hybridization technique that can detect low-abundance RNA within tissue samples,^30^ enabling researchers to both quantify and visualize the location of target RNA transcripts within the tissue.^30^ Therefore, the aim of this study was to detect the level and location of the HCMV *lncRNA4.9* gene product within the breast tumor microenvironment of formalin-fixed paraffin-embedded (FFPE) ER/PR positive (ER+/PR+), HER2-overexpressing (HER2+), and TNBC (ER−/PR−/HER2−) samples. HCMV immunoglobulins (IgM, IgG) were assayed in serum samples from the study cohort, and standard immunostaining techniques were used to visualize tissue architecture and macrophage populations within the breast tissue samples.

## Patients and Methods

### Study Cohort

This study used FFPE breast tumor samples from women with early stage (stage 1–3) breast cancer (*n*=75), comprising three clinically relevant breast cancer subtypes: estrogen receptor/progesterone receptor positive (ER+/PR+), Human Epidermal Growth Factor Receptor 2 overexpressing (HER2+), and triple receptor negative (ER−/PR−/HER2−; also known as triple-negative breast cancer, TNBC). Samples were collected via He Taonga Tapu Cancer Society Tissue Bank Christchurch from treatment naïve patients who received adjuvant (post-surgery) treatment. The study cohort was enriched to increase the number of TNBC tumors in the study cohort to ~33% compared with the expected ~10% to 20% in the breast cancer patient population.^31^ Normal breast tissue samples obtained from women having mammary reduction surgery or for prophylactic reasons as BRCA1/2 carriers with no known breast cancer were included as a control group for this study (*n*=5). Ten consecutive tissue sections (4 μm) were cut from each FFPE breast tissue sample (*n*=75 tumor, *n*=5 normal). Whole-slide FFPE tissue sections with regions of both histologically normal and breast tumor tissue were used in this study. Clinicopathological and patient follow-up data was supplied by He Taonga Tapu Cancer Society Tissue Bank and was blinded to the researchers until final analysis. All patients gave informed written consent for use of their samples. Ethics approval was obtained from the University of Otago Human Ethics Committee (Ethics approval #H21/036) and permission to use banked samples was given by the Canterbury Tissue Bank Board.

### Serology

To establish HCMV serological status, a serum sample from each patient was assayed by Canterbury Health Laboratories for HCMV IgG (*n*=76 samples available) and IgM (*n*=64 samples available) seropositivity using standard enzyme immunoassays (Euroimmun, Lübeck, Germany).

### RNA In Situ Hybridization (RNAscope)

To detect the presence of HCMV *lncRNA4.9* within breast tumor tissue, RNA in situ hybridization was conducted on whole-slide FFPE tissue sections and cell line control sections using the RNAscope 2.5 HD Assay—RED detection kit (Advanced Cell Diagnostics, Hayward, CA), as per the manufacturer’s protocol. The RNAscope target probe V-HCMV-RNA4.9 (Catalog number #569411, Appendix Fig. 1), positive control probe Hs-*PPIB* (Catalog number #313901-C2), and negative control probe bacterial *dapB* (Catalog number #310043-C2) were selected from the Advanced Cell Diagnostics RNAscope probe catalog. Briefly, FFPE sections (4 µm) were placed onto glass microscope slides and then deparaffinized in a series of xylene and 100% ethanol steps and incubated with hydrogen peroxide to block endogenous peroxidase activity. Manual target retrieval was performed as per manufacturer’s instructions, and the slides left to dry at room temperature overnight. Slides were treated with Protease Plus before hybridization of RNAscope target and control probes. Hybridization of RNAscope probes involved dispensing four to six drops (approximately 150 µl) from a ready-to-use bottle onto the slide, enough to cover the tissue section. Slides were then placed and covered in a HybEZ Humidity Control Tray and incubated at 40C for 2 hr in a HybEZ Oven. Amplification of RNAscope probes was conducted as per manufacturer’s instructions in six consecutive hybridization steps using reagents AMP1-AMP6, before application of the RNAscope Fast-RED solution for color development and detection of the RNA target probes. Slides were then counterstained with Gill’s hematoxylin (Richard-Allan Scientific, Kalamazoo, MI) and left to dry before applying mounting media and coverslips. Modifications to the manufacturer’s protocol included performing three xylene washes for 5 min each during the deparaffinization procedure, increasing the incubation period of the AMP5 reagent from 30 min to 1 hr, and submerging the slides 20 times in 50:50 hematoxylin to counterstain tissue sections. Manual assessment of tissue sections was conducted, blinded to clinicopathological data, using a bright field microscope (Zeiss Axiolmager.Z1 Universal Research Microscope). Slides were determined to be positive for mRNA expression of the target probes if red punctuate dots were seen within cells and tissue.

#### Control Array

**Figure 1.**
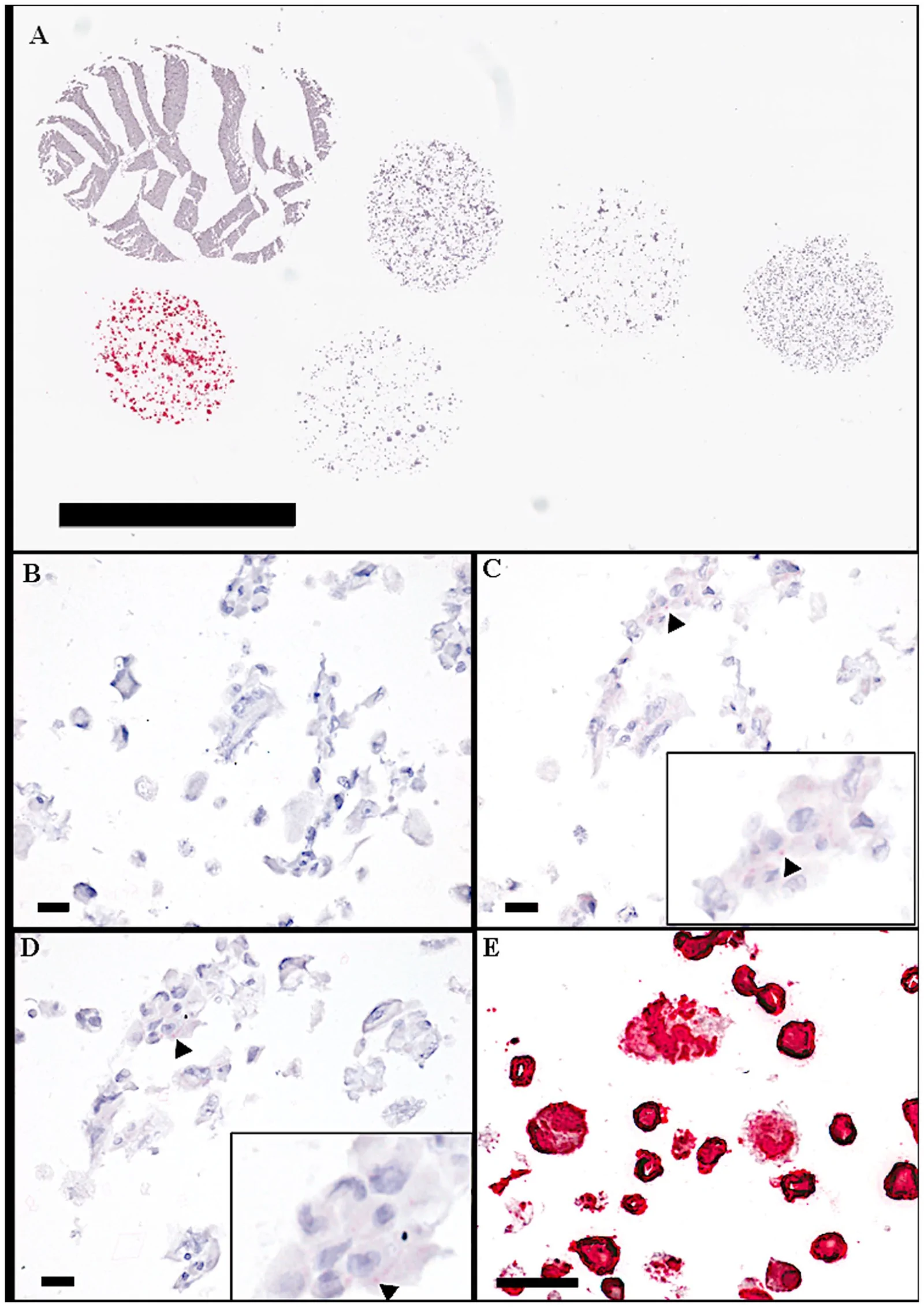
RNAscope *lncRNA4.9* target probe on control array. To demonstrate specificity of the *lncRNA4.9* target probe, RNAscope was conducted on four serial sections of a control array (Fig. 1A). The control array consisted of FFPE heart muscle (orientation control) and cores of FFPE epithelial cells infected with five specific human viruses (Human SV1, Human SV2, SV40/Polyoma, Human CMV, and Human EBV). Each array section was treated with a different RNAscope probe; the HCMV *lncRNA4.9* target probe, bacterial *dapB* negative control probe, *PPIB* positive control probe, and *UBC* positive control probe. (A) Whole section of the control array hybridized with the HCMV *lncRNA4.9* target probe displaying red punctate hybridization signals only in the HCMV core (1× magnification; scale bar 2 mm). (B) *dapB* negative control at 40× magnification (scale bar 20 µm). (C) *PPIB* positive control at 40× magnification (scale bar 20 µm). (D) *UBC* positive control at 40× magnification (scale bar 20 µm). (E) HCMV-infected cell core probed for HCMV *lncRNA4.9* at 40× magnification (scale bar 50 µm). Arrowheads show positive signals (red) for each RNAscope target probe.

RNAscope HCMV *lncRNA4.9* target probe specificity was determined using a human cell control array—virus (Zytomed Systems, Berlin, Germany). The cell control array consists of an array of five FFPE human epithelial cell cores each infected with a specific human virus (Human SV1, Human SV2, SV40/Polyoma, Human CMV, and Human EBV), as well as an FFPE heart muscle core to facilitate orientation of the array (Fig. 1A and Appendix Fig. 2). Four 5-µm-thick serial sections were cut from the array and placed onto glass microscope slides (Trajan Scientific and Medical, Ringwood, Australia). RNA in situ hybridization was performed using RNAscope probes HCMV *lncRNA4.9*, *PPIB* (positive control), *UBC* (positive control), and a bacterial *dapB* negative control as per the RNAscope 2.5 HD Assay—RED detection kit protocol (Advanced Cell Diagnostics, Hayward, CA).

#### Tissue Assessment and Semi-Quantitative RNAscope Scoring

Manual assessment of whole-slide FFPE tissue sections was conducted by a researcher (G.T.) using a bright-field microscope (Zeiss Axiolmager.Z1 Universal Research Microscope) with supervision by a senior medical laboratory scientist (A.M.-B.) and guidance from a specialist breast pathologist (B.B.). All researchers were blinded to the clinicopathological data during tissue assessment. Whole slides were digitally scanned at 40× magnification using an Aperio Imaging System (Lecia Biosystems Pty Ltd, VIC, Australia) by OMNI Histology services (University of Otago, Dunedin). Whole-slide images were manually assessed at 40× magnification using Aperio Imagescope—Pathology Slide Viewing Software v12.4.6.5003 (Leica Biosystems Pty Ltd, VIC, Australia).

The level and location of HCMV *lncRNA4.9* transcripts was recorded for each case. Semi-quantitative assessment of HCMV *lncRNA4.9* expression was classified as either low/medium/high expression based on the number of cells displaying HCMV *lncRNA4.9* probe signal within a tissue area, the number of probe signals detected in each cell, the intensity of probe signal, the number of areas within the tissue that were positive for target probe signal, and the magnification at which the probe signal could be visualized (Table 1).

**Table 1.** Classification Criteria of RNAscope Positive Samples.

Low	Small numbers of positive cells (<20% of cells), each cell with a low amount of probe detected (1–3 dots per cell), very light staining with the probe, one or two areas with positivity and higher magnification required to observe the probe.
Mid	Higher number of positive cells (20–50% of cells), more probes detected within each cell (4–10 dots per cell), higher intensity of probe signal, larger and/or more areas of positivity and a slightly lower magnification required to visualize the probes.
High	Large numbers of cells (>50% of cells) with bright positivity, most of the area within each cell positive (>10 dots per cell), larger areas of positivity, low magnification required to visualize the probe.
Localized	Distinct area(s) of positivity within observed area of a tissue sample.
Diffuse	Sporadic areas of positivity throughout tissue sample.

#### Quantification of RNAscope Probe Signal

To quantify RNAscope probe hybridization signals, whole-slide images were analyzed using the QuPath 0.3.2 open-source software for digital pathology image analysis.^32^ Images of each tissue section hybridized with the RNAscope target probe were uploaded to the QuPath software, and bright-field image criteria selected. The software was trained to identify RNAscope target probe hybridization signals by selecting areas of positive signal in each region of interest (ROI). This was achieved in the software by training a pixel classifier to set pixel thresholds for positive staining for RNA probe signals (red punctuate dots and clusters) from annotation examples of positive RNA probe signal staining. Similarly, cells counted within the ROI were detected using the positive cell detection command and setting the cell nuclei detection with the hematoxylin stain intensity. For each sample, five representative ROIs (250 μm × 300 μm) of tumor tissue were analyzed at 40× magnification. In these representative ROIs, all cells and RNAscope probe signals (discrete “spots” and “clusters” of probe signal) were counted by the software. Counts of both the number of cells and RNAscope probe signals were used to calculate the number of *lncRNA4.9* transcripts per cell.

### Immunohistochemistry

Immunohistochemistry (IHC) was conducted to visualize macrophage populations within breast tissue samples using the DAKO Envision+ Dual Link System-HRP (DAB+) (Dako Denmark A/S, Glostrup, Denmark). Antibody dilution was optimized using an FFPE control section containing normal spleen, liver, skin, and appendix tissue obtained from He Taonga Tapu Cancer Society Tissue Bank. Analysis of the pan-macrophage surface antigen CD68 was conducted using a monoclonal mouse anti-human CD68 antibody (Catalog number ^#^M0876, Dako Denmark A/S, Glostrup, Denmark). Human breast tissue controls for this experiment included a positive control for CD68, a negative phosphate-buffered saline (PBS) control, and a mouse IgG isotype control (Catalog number ^#^31903, Invitrogen, Thermofisher, Rockford, IL). To perform antigen retrieval, slides were deparaffinized and placed within a pressure cooker containing near-boiling sodium citrate buffer (pH 6.0) for 3 min. Slides were left to cool in solution for 40 min before being washed three times for 5 min in Tris-buffered saline with Tween (TBST). A hydrophobic barrier was drawn around the tissue sections before incubation of the slides with DAKO dual exogenous enzyme for 10 min. The anti-CD68 primary antibody was optimally diluted to 1:500 in 1% bovine serum albumin (BSA) + Phospate-buffered saline with Tween (PBST) and applied to each breast tissue slide and the positive control. PBS diluted 1:500 with 1% BSA + PBST was applied to the negative control, and mouse IgG diluted 1:500 with 1% BSA + PBST was applied to the isotype control. All sections were incubated within a humidity chamber at room temperature for 30 min. Slides were washed and then the Labelled Polymer-HRP Rabbit/Mouse reagent was applied and incubated within the humidity chamber at room temperature for 30 min. DAB+ chromogen working reagent was applied and left to incubate for 10 min at room temperature inside the humidity chamber. Slides were counterstained with Gill’s hematoxylin and dehydrated before mounting media and coverslips were applied. Slides were examined and assessed using a bright-field microscope (Zeiss Axiolmager.Z1 Universal Research Microscope).

#### Semi-Quantitative Assessment of CD68 Immunohistochemistry

For each breast tissue sample, two representative areas were selected at 20× magnification (this allowed the reviewers to ascertain the localization of positivity relative to tissue architecture while permitting high cellular resolution). Each representative ROI selected was given a semi-quantitative score for anti-CD68 immunostaining based on the proportion of cells within the area that stained positive for the CD68 macrophage marker. The overall score for each sample was determined by averaging the score from the two areas. Scoring criteria was classified as follows: 0 (negative), 1+ (≤10% of cells within the area stained), 2+ (10–30% of cells stained), and 3+ (>30% of cells within the area stained).

### Co-Localization of CD68 Protein and HCMV lncRNA4.9 Expression

To determine any potential relationship between the presence of CD68-positive macrophages and HCMV *lncRNA4.9*, whole-slide images of anti-CD68 immunostained breast tissue sample sections were visually aligned, side-by-side to the corresponding tissue regions in consecutive sections that were RNAscope positive for HCMV *lncRNA4.9*. Confirmation of the co-localization was performed by the reviewers and pathologist.

### Statistical Analysis

Chi-square statistical tests were performed to analyze RNAscope, serological, and IHC data generated from this study. One-way analysis of variance tests were performed to test the statistical significance of RNAscope quantification results between each breast cancer subtype. Statistical analysis of data was performed using the GraphPad Prism 9 program (GraphPad Software, San Diego, CA).

## Results

### Study Cohort

In the study cohort (*n*=80 total, *n*=75 breast tumor, *n*=5 normal breast tissue), 76% (*n*=60 breast tumors, *n*=1 normal breast tissue) of women were over 50 years of age (Table 2). The study cohort was enriched for TNBC, so samples in the study cohort comprised a third of each clinical subtype (ER+/PR+ *n*=25, HER2 + *n*=24, TNBC *n*=26) as well as normal breast tissue samples (*n*=5) from mammary reduction surgeries. Of the 75 breast tumor samples, 64 were grade 3, with 52 tumors greater than 21 mm in size. Histological subtypes included invasive ductal carcinoma (*n*=73) and apocrine tumors (*n*=2), with the majority of tumors exhibiting a single, localized tumor foci (Table 2).

**Table 2.** Clinicopathological Data for the Study Cohort Including *lncRNA4.9* RNAscope Status.

Total Cohort (*n*=80)	*n* (%)	RNAscope +ve	RNAscope −ve
Age			
<50 breast tumor	15 (19%)	9 (11%)	6 (7%)
≥50 breast tumor	60 (75%)	36 (45%)	24 (30%)
<50 normal breast tissue	4 (5%)	2 (2%)	2 (2%)
≥50 normal breast tissue	1 (1%)	0 (0%)	1 (1%)
Clinical subtype			
ER+/PR+	25 (31%)	18 (22%)	7 (9%)
HER2+	24 (30%)	14 (18%)	10 (13%)
TNBC	26 (33%)	13 (16%)	13 (16%)
Normal	5 (6%)	2 (2%)	3 (4%)
**Tumor samples (*n*=75)**			
Tumor size (mm)			
0–20	23 (31%)	13 (17%)	10 (13%)
≥21	52 (69%)	32 (43%)	20 (27%)
Tumor grade			
1	2 (3%)	0 (0%)	2 (3%)
2	9 (12%)	4 (5%)	5 (7%)
3	64 (85%)	41 (55%)	23 (30%)
Histological type			
Ductal	73 (97%)	43 (57%)	30 (40%)
Apocrine^a^	2 (3%)	2 (3%)	0 (0%)
Tumor foci			
Localized	69 (92%)	42 (56%)	27 (36%)
Multifocal	6 (8%)	3 (4%)	3 (4%)
**Serology**			
**HCMV IgG** ^b^ **(*n*=76)**			
Positive	33 (43%)	20 (26%)	13 (17%)
Negative	42 (55%)	24 (32%)	18 (24%)
Equivocal	1 (1%)	1 (1%)	0 (0%)
**HCMV IgM** ^c^ **(*n*=64)**			
Positive	26 (41%)	13 (20%)	13 (20%)
Negative	33 (52%)	25 (39%)	8 (13%)
Equivocal	5 (8%)	3 (5%)	2 (3%)

Abbreviations: ER+/PR+, estrogen receptor positive/progesterone receptor positive status; HER2+, human epidermal growth factor 2 receptor status; TNBC, triple-negative receptor status; +ve, positive; −ve, negative.

a After review of hormone receptor status, both apocrine carcinoma cases were classified as TNBC and analyzed together with TNBC cases.

b HCMV IgG serology data available for *n*=76 serum samples; *n*=71 breast cancer and *n*=5 normal breast reduction surgery.

c HCMV IgM serology data available for *n*=64 serum samples; *n*=60 breast cancer and *n*=4 normal breast reduction surgery.

### Serology

To determine the HCMV serological status of the study cohort, serum samples were tested for the presence of HCMV IgG (*n*=76 total, *n*=71 breast cancer, *n*=5 normal, *n*=4 not available for testing), and HCMV IgM (*n*=64 total, *n*=60 breast cancer, *n*=4 normal, *n*=16 not available for testing).

Serology testing for HCMV IgG showed 43% of samples tested positive (*n*=32 breast cancer, *n*=1 normal), 55% of samples were seronegative (*n*=38 breast cancer, *n*=4 normal), and one sample gave equivocal results (Table 2). Similarly, serology testing for HCMV IgM identified 41% serum samples were seropositive (*n*=24 breast cancer, *n*=2 normal) and 52% samples (*n*=31 breast cancer, *n*=2 normal) were seronegative, with one sample equivocal (Table 2).

For IgG serology, women with ER+/PR+ breast cancer had the highest number of IgG-positive serum samples (18/24, 75%), compared with 41% (9/22) in the HER2+ group and 20% (5/25) in the TNBC group (Chi-square test; *p*=0.0004). Of the normal breast samples, only one serum sample was positive for HCMV IgG, with all others identified as seronegative (Appendix Table 1). HCMV IgM seropositivity was higher in serum samples from women with HER2+ overexpressing (10/19, 53%) and TNBC breast tumors (11/23, 48%) compared with those with ER+/PR+ (3/18, 17%) tumors. Serum HCMV IgM status was positive in two of the four normal breast tissue samples available for analysis (Appendix Table 2).

### RNAscope

For RNAscope, an FFPE control array (Fig. 1; Appendix Fig. 2) with tissue cores consisting of heart muscle and cell lines infected with human viruses, including HSV1, HSV2, SV40/Polyoma, HCMV, and EBV, was used to confirm the specificity of the HCMV *lncRNA4.9* target probe (Fig. 1A). Four control array sections were hybridized with different RNAscope probes, including bacterial *dapB* negative control, *PPIB* positive control, *UBC* positive control, and the HCMV *lncRNA4.9* target probe (Fig. 1B–E).

No probe signal was detected in the control array section hybridized with the bacterial *dapB* negative control probe (Fig. 1B). Hybridization signals were detected in both the *PPIB* and *UBC* positive controls, with signals best visualized at 40× magnification (Fig. 1C and D insets). Within the control array section hybridized with the HCMV *lncRNA4.9* target probe, only the HCMV-infected cell core displayed bright and punctuate target probe hybridization signals, with no signaling in any other cell core infected with other human viruses (Fig. 1A and E).

**Figure 2.**
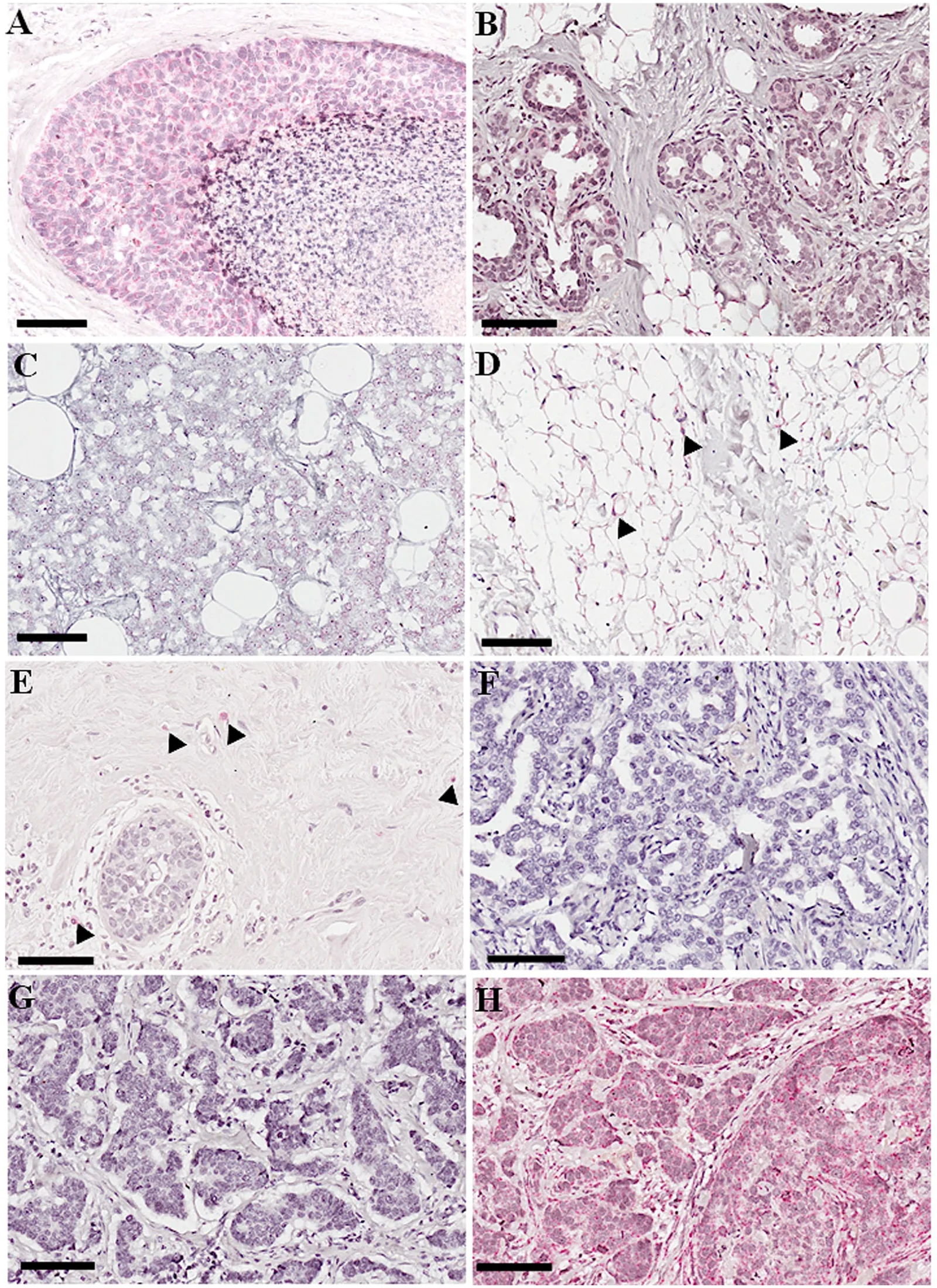
Representative images of HCMV *lncRNA4.9* RNAscope probe hybridization signals observed within normal breast tissue and breast tumor samples. RNAscope was performed on sections from normal breast tissue and breast tumor samples to detect HCMV *lncRNA4.9* probe, as well as positive *PPIB* probe and negative *dapB* probe controls. Target probe hybridization signals were observed within the following breast tissues and cell types: (A) Ductal carcinoma in situ (DCIS), (B) breast lobules and ducts, (C) invasive tumor tissue, (D) adipose tissue, and (E) individual cells within the stroma of breast tissue. (F) Breast tumor tissue sample classified as RNAscope negative. (G) *dapB* negative control probe in breast tumor tissue, and (H) *PPIB* positive control probe in breast tumor tissue. Arrowheads show punctate red staining of HCMV *lncRNA4.9* probe hybridization signal. All images 20× magnification, scale bar 100 µm.

Of the 80 breast tissue samples tested (breast tumor *n*=75, normal breast *n*=5), 47 breast tissue samples exhibited hybridization signals with the HCMV *lncRNA4.9* target probe (*n*=45 breast tumor, *n*=2 normal) (Table 2). HCMV *lncRNA4.9* probe hybridization signals were detected in invasive tumor tissue (*n*=27), breast ducts (*n*=16), adipose tissue (*n*=15), stroma (*n*=14), and associated foci of ductal carcinoma in situ (DCIS) (*n*=13) (Appendix Table 3; Fig. 2), with over half (*n*=26) of the HCMV *lncRNA4.9*-positive breast samples showing *lncRNA4.9* hybridization signals in more than one breast tissue type (Appendix Table 3 and Appendix Fig. 3). Invasive carcinoma cells were more likely to have HCMV *lncRNA4.9* transcripts present compared with normal breast epithelial cells or cells within foci of DCIS (RR=1.68, 95% CI: 1.08, 2.9, *p*=0.025). Within the two normal breast tissue samples that were classified as positive, *lncRNA4.9* hybridization probe signal was only detected at low levels within individual cells of the stroma (≤16 positive cells per tissue section; data not shown).

**Figure 3.**
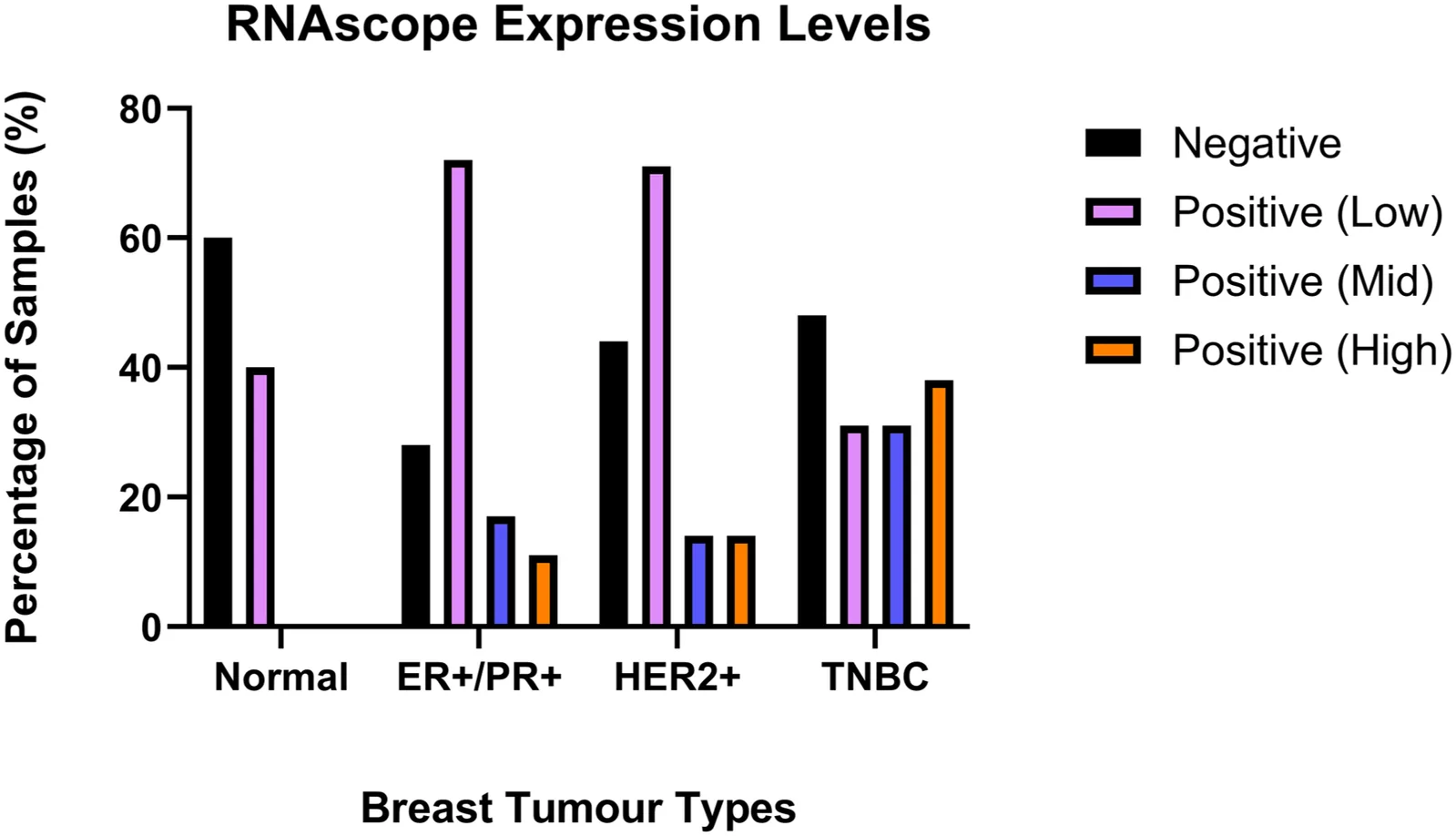
RNAscope assessment of HCMV *lncRNA4.9* expression levels in breast tumor subtypes. RNAscope was conducted on whole-slide FFPE sections of normal breast tissue and breast tumor samples (ER+, HER2-overexpressing, TNBC) from the study cohort to detect HCMV *lncRNA4.9* expression. Tissue samples were classified as having negative (black), low- (pink), mid- (blue), or high- (orange) levels of *lncRNA4.9* expression based on semi-quantitative assessment of the number of *lncRNA4.9* positive cells per region and the number of *lncRNA4.9* hybridization signals per cell (see Table 1 for classifications).

No statistical differences were observed in the number of samples positive for *lncRNA4.9* hybridization probe signal among the breast cancer clinical subtypes studied (Table 2; Chi-square test; *p=*0.33). Eighteen (22%) ER+/PR+ breast tumor samples were classified as positive and seven samples (9%) classified as negative for *lncRNA4.9* hybridization probe signal (Table 2). Similarly, 14 (18%) HER2+ overexpressing breast tumor samples were classified as positive and 10 samples (13%) as negative. For TNBC, there was an equal split, with 13 (16%) samples classified as positive and 13 samples (16%) classified as negative for *lncRNA4.9* hybridization probe signal (Table 2, Fig. 3).

Similarly, no significant differences were observed in the number of *lncRNA4.9* transcripts per cell among the breast cancer clinical subtypes studied. Quantification was conducted using the QuPath software package to assess five regions of interest (ROI) within each tissue sample. First, the average number of cells were assessed across the five ROIs in each sample. For both ER+/PR+ and TNBC, the majority of breast tumor samples had an average of 500–650 cells per ROI, whereas cell numbers tended to be lower in HER2+ overexpressing samples with an average of 350–500 cells per ROI assessed (Appendix Fig. 4A). Neither the number of individual *lncRNA4.9* transcripts per cell nor the number of *lncRNA4.9* probe signal clusters per cell was significantly different between breast cancer subtypes. Although *lncRNA4.9* probe signals were detected in all five normal breast samples, only one sample had more than one *lncRNA4.9* transcript per cell (Appendix Fig. 4B and C).

Of note, a greater proportion of TNBC samples showed mid- to high levels of HCMV *lncRNA4.9* expression compared with ER+ or HER2-overexpressing samples (Fig. 3). Tumor samples were classified as having low, mid-, or high levels of *lncRNA4.9* expression based on the number of *lncRNA4.9*-positive cells per ROI and the number of *lncRNA4.9* hybridization signals per cell (Table 1). The majority of *lncRNA4.9*-positive samples within the ER+/PR+ and HER2 overexpressing subtypes had low *lncRNA4.9* expression levels [*n*=13/18 (72%) and *n*=10/14 (71%), respectively], with small numbers of *lncRNA4.9* probe positive cells (<20%) and a low number of probe signals detected per cell (1–3 signals per cell) (Table 1, Fig. 3). The TNBC subtype group had only *n*=4/13 (31%) of positive samples with low *lncRNA4.9* expression levels. Two of the five normal breast tissue samples were identified as RNAscope positive, with both samples exhibiting low expression levels for the *lncRNA4.9* target probe (Fig. 3). Overall, fewer samples for the ER+/PR+ and HER2 overexpressing subtypes were classified as having mid expression levels of the RNAscope *lncRNA4.9* probe [*n*=3/18 (17%) and *n*=2/14 (14%), respectively], compared with *n*=4/13 (31%) of TNBC samples. Similarly, both ER+/PR+ and HER2+ subtype groups each had two tumor samples with high expression for *lncRNA4.9* probe signal [*n*=2/18 (11%) and *n*=2/14 (14%), respectively], whereas the TNBC subtype had five (38%) breast tumor samples with high expression of *lncRNA4.9* probe signal (Fig. 3; See Table 1 for classification of expression). These were detected in invasive cancer cells, DCIS, and adipose tissue.

### Immunohistochemistry

The pan-macrophage marker anti-CD68 exhibited consistent immunohistochemical staining of high intensity within positive control tissues including spleen, liver, skin, and appendix, with low non-specific background signal (Appendix Fig. 5A). No staining was detected in sections stained using negative controls that replaced the primary antibody with PBS solution alone or the equivalent mouse IgG isotype control antibody (Appendix Fig. 5B and C, respectively).

**Figure 4.**
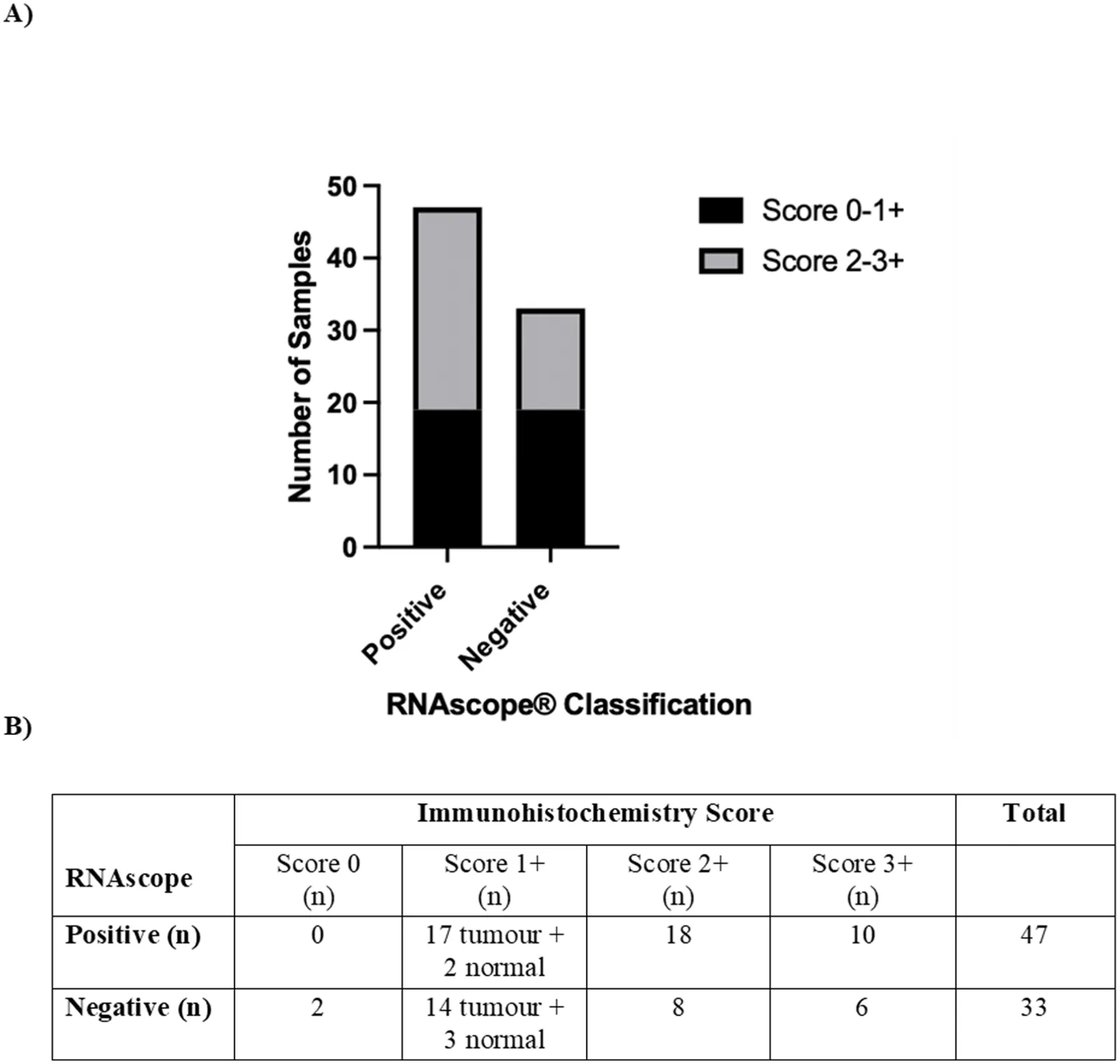
Comparison of CD68+ immunohistochemistry score with RNAscope positive and negative samples. IHC was used to immunostain each breast tissue sample (*n*=75 tumor, *n*=5 normal breast) for the pan-macrophage marker CD68, and samples were scored according to the number of cells within a representative area that were CD68^+^ (0, 0%; 1^+^, ≤10%; 2^+^, 10–30%; 3^+^, >30%). The score for each sample was compared with the RNAscope HCMV *lncRNA4.9* analysis classification (positive vs negative). (A) Graph showing IHC staining score and RNAscope positive or negative status within samples. (B) Table of the IHC staining score of RNAscope positive and negative samples. No significant differences were observed (Chi-square test, *p=*0.13).

Only two samples within the study cohort displayed no anti-CD68 staining (IHC score = 0), and both were also classified as HCMV *lncRNA4.9* negative by RNAscope (Fig. 4B). All five normal breast tissue samples and 31 breast tumor samples received an IHC score of 1+. For the breast tumor samples with IHC score of 1+, 17 were classified as HCMV *lncRNA4.9* positive and 14 were HCMV *lncRNA4.9* negative (Fig. 4B). Twenty-six breast tumor samples received an IHC score of 2+ (18 HCMV *lncRNA4.9* positive, eight HCMV *lncRNA4.9* negative), and 16 breast tumor tissue samples received an IHC staining score of 3+ (10 HCMV *lncRNA4.9* positive, six HCMV *lncRNA4.9* negative) (Fig. 4B). Although HCMV *lncRNA4.9* positive breast tissue samples had double the number of high (score 2–3+) IHC scores compared with the HCMV *lncRNA4.9* negative samples, no statistical significance was observed (Fig. 4A and B) (Chi-square test; *p=*0.1305).

**Figure 5.**
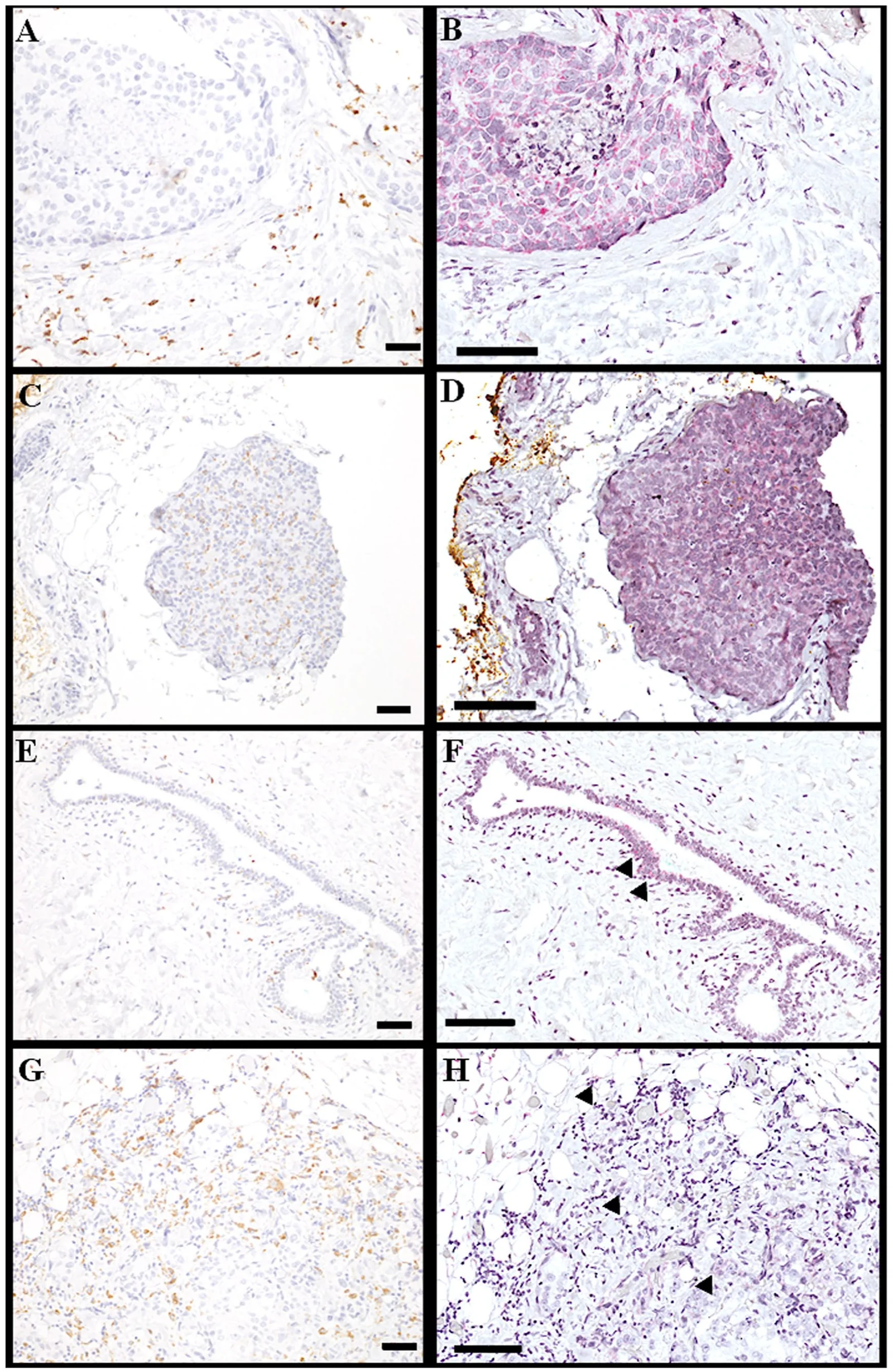
Comparison of breast tumor samples analyzed by IHC for CD68+ cells (left panels) and RNAscope for HCMV *lncRNA4.9* transcripts (right panels). Breast tumor sample whole-slide sections showing: (A) ductal carcinoma in situ (DCIS) and surrounding stroma immunostained for CD68 (brown), and (B) consecutive section showing localization of HCMV *lncRNA4.9* probe hybridization signal in DCIS and stroma (red); (C) area of isolated tumor cells immunostained for CD68 (brown), and (D) consecutive section showing localization of HCMV *lncRNA4.9* probe hybridization signal in isolated tumor cells (red); (E) breast duct and the surrounding stroma immunostained for CD68 (brown), and (F) consecutive section analyzed by RNAscope for HCMV *lncRNA4.*9 hybridization probe signal (red). (G) Invasive tumor cells and adipose tissue with CD68-positive IHC staining (brown), and (H) consecutive section analyzed by RNAscope for HCMV *lncRNA4.9* hybridization probe signal (red). Arrowheads show punctate red staining of HCMV *lncRNA4.9* probe hybridization signal. Photomicrographs at 20× magnification, all IHC images scale bar 50 µm, all RNAscope images scale bar 100 µm.

When comparing HCMV *lncRNA4.9* RNAscope positive breast tumor tissue sections to the consecutive anti-CD68 IHC stained section, the location of anti-CD68 immunostaining varied in relation to the location of HCMV *lncRNA4.9* target probe signal (Fig. 5). In breast tissue samples that had HCMV *lncRNA4.9* hybridization signal localized within DCIS (Fig. 5B), anti-CD68 immunostaining occurred in the surrounding stroma but was not co-localized with HCMV *lncRNA4.9* probe hybridization signal within the DCIS site (Fig. 5A and B). Similarly, anti-CD68 staining was not observed to co-localize with HCMV *lncRNA4.9*-positive ductal epithelial cells but was present in the surrounding stroma (Fig. 5E and F). However, in isolated tumor cells (Fig. 5C and D) and at the invasive tumor margin, high numbers of CD68+ cells were observed and were co-localized within the same area of HCMV *lncRNA4.9* positivity (Fig. 5C, D, G, H).

## Discussion

To our knowledge, this is the first study to use the RNA in situ hybridization technique RNAscope to demonstrate the presence of a gene product of HCMV (*lncRNA4.9*) in pre-invasive DCIS and invasive ductal carcinoma, and in different cell types within the tumor microenvironment of ER+/PR+, HER2-overexpressing, and TNBC tumor samples. Although this study did not provide sufficient evidence to corroborate the hypothesis that late exposure to a common virus such as HCMV may cause some breast cancers,^20^ it demonstrated the widespread presence of HCMV *lncRNA4.9* in FFPE human breast tissue samples and indicated recent HCMV exposure may occur more often in HER2+ and TNBC subtypes.

### Serological Status of the Study Cohort

Serology tests that detect HCMV antibodies can be used to infer lifetime exposure to HCMV, with a positive HCMV IgG showing exposure to HCMV (past or recent), and positive HCMV IgM indicating an active or recent infection (primary, reactivation, or reinfection).^33^ A previous study by our group, using a similar local patient cohort (*n*=70), detected HCMV IgG within 70% of the patient samples, and noted that younger women were generally more seropositive.^25^ The current study also indicated widespread HCMV seropositivity, with 43% of tested samples positive for HCMV IgG and 41% of tested samples positive for HCMV IgM. In the current cohort, results also indicated differences among breast cancer subtypes, with significantly more samples seropositive for IgG from women with ER+/PR+ breast tumors than from women with HER2-overexpressing or TNBC tumors. In contrast, IgM seropositivity was higher in HER2-overexpressing and TNBC samples than ER+/PR+ samples. These findings suggest that recent infection or reactivation of infection is more commonly associated with HER2-overexpressing and TNBC cancer subtypes, and less likely associated with ER+/PR+ cancers. These observations corroborate previous findings by Herbein et al,^26,34,35^ who demonstrated an association between HCMV and TNBC tumors in human breast cancer tissue.^26^ However, the small number of samples in each clinical subtype and unavailability of serology results for a number of samples is a limitation of the current study that means further investigation is required to establish a link between late HCMV infection (or reactivation of infection) and breast cancer development in non-estrogen-responsive breast tumors.^20^

### RNAscope for Detection of HCMV Gene Product lncRNA4.9

Most previous studies have used polymerase chain reaction to assay HCMV gene products in breast tumor tissue.^23,25^ This has led to inconsistent results, with studies targeting HCMV Intermediate Early (IE) genes (e.g. IE1/UL123) most likely to report HCMV detection.^23,36^ Kumar et al.^26^ demonstrated some strains of HCMV are capable of oncogenesis by experimentally transforming human mammary epithelial cells (HMECs) with a clinical isolate of HCMV (HCMV-DB). Although they did not detect the full viral genome in the transformed HMECs, they found the *lncRNA4.9* gene sequence was detected in the transformed HMECs and in the triple-negative breast xenograft tumors that developed in immunodeficient mice injected with the transformed HMECs. Moreover, genomic sequence of *lncRNA4.9* was also detected in biopsies of women with TNBC.^26,37^ Previous studies have shown *lncRNA4.9* may have several contrasting functions during HCMV infection. For example, it interacts with polycomb repression complex 2 (PCR2) to mediate transcriptional repression of the major immediate-early promoter during HCMV latency,^29^ and it is localized to viral DNA replication sites during viral replication and growth.^28^

For these reasons, we used the sensitive and specific RNA in situ hybridization technique RNAscope to detect the HCMV gene product (*lncRNA4.9*) in FFPE breast tumors. RNAscope amplifies individual target probe hybridization signals (i.e. *lncRNA4.9* transcripts) and allows visualization of their number, probe hybridization signal intensity and location within the tissue architecture. We used *lncRNA4.9* RNAscope hybridization probes commercially available from Advanced Cell Diagnostics and conducted an array of controls to demonstrate probe specificity to target human CMV *lncRNA4.9*. However, as the RNAscope hybridization probe sequences remain proprietary to Advanced Cell Diagnostics, we cannot state with certainty that the HCMV *lncRNA4.9* probes used in the current study detect oncogenic high-risk strains of HCMV.^26^

We have demonstrated HCMV *lncRNA4.9* in both normal breast and breast tumor tissue (albeit at very low expression in normal breast tissue). Over half (60%) of breast tissues studied exhibited HCMV *lncRNA4.9* probe hybridization signal, and in the majority of these positive tumor samples, HCMV *lncRNA4.9* transcripts were detected in several different breast cell types and tissue compartments. Specifically, *lncRNA4.9* probe hybridization signal was observed in normal epithelial cells of breast ducts, DCIS, invasive ductal carcinoma, breast adipocytes, and individual cells within the tumor stroma. Although *lncRNA4.9* probe positivity was most common in regions containing invasive ductal carcinoma cells, our findings support widespread HCMV infection in over half the study cohort from the earliest stages of breast cancer development, potentially facilitating both oncogenesis and oncomodulation.^26,27^

Our study is one of the few that have accounted for breast tumor subtype/receptor status, allowing us to address the possibility that association with HCMV is strongest in hormone receptor negative breast cancers. Although we enriched the TNBC samples to 33% of cohort (compared with the expected 10–20% in the general population),^31^ we did not see any significant differences in the number of tumor samples expressing *lncRNA4.9* transcripts among the breast cancer subtypes studied. However, of the HCMV *lncRNA4.9*-positive samples, TNBC samples had the greatest number of samples with mid-high levels of HCMV *lncRNA4.9* expression, with more than 50% of cells showing bright positivity and more than 10 *lncRNA4.9* probe signals per cell. This corroborates previous studies that observed the presence of HCMV in non-estrogen-responsive TNBC.^26,37^

Although HCMV can infect many cell types, it shows particular tropism for antigen-presenting cells of the myeloid lineage, including monocytes, macrophages, and dendritic cells.^38^ HCMV encodes several molecules that block antigen presentation by major histocompatability complex and CD1 proteins,^17^ and previous studies have demonstrated that HCMV-infected tumor-associated macrophages modulate the breast tumor microenvironment by repressing immune activation and favoring a permissive, pro-tumoral microenvironment.^38,39^ In the current study, immunohistochemical analysis showed almost twice the number of CD68+ cells in HCMV *lncRNA4.9* positive compared with negative samples. We also observed distinct patterns in the location of CD68+ cells in relation to HCMV *lncRNA4.9*-positive cells. For example, where HCMV *lncRNA4.9*-positive probe signals were present within foci of DCIS or in normal epithelial cells in breast ducts, CD68+ cells were detected in the surrounding stroma. In contrast, at the invasive margin, CD68+ cells were numerous and co-localized with HCMV *lncRNA4.9*-positive epithelial cells and cancer-associated adipocytes. Future studies are needed to further characterize the immunophenotype of these CD68+ cells and determine whether they are immune effector or tumor-promoting cells.

In conclusion, we have used RNAscope RNA in situ hybridization to demonstrate that HCMV *lncRNA4.9*-positive cells are present in normal breast tissue, DCIS, and invasive ductal carcinoma, and that HCMV *lncRNA4.9* can be detected in different cell types (e.g. epithelial cells, adipocytes, and stromal cells) within the breast tumor microenvironment. Future studies in large, well-characterized cohorts should investigate whether enumeration of HCMV *lncRNA4.9* transcripts in breast cancer cells may provide a clinically useful and easily implemented prognostic marker for a subset of breast cancers.

## Figures and Tables

**Figure A1.**
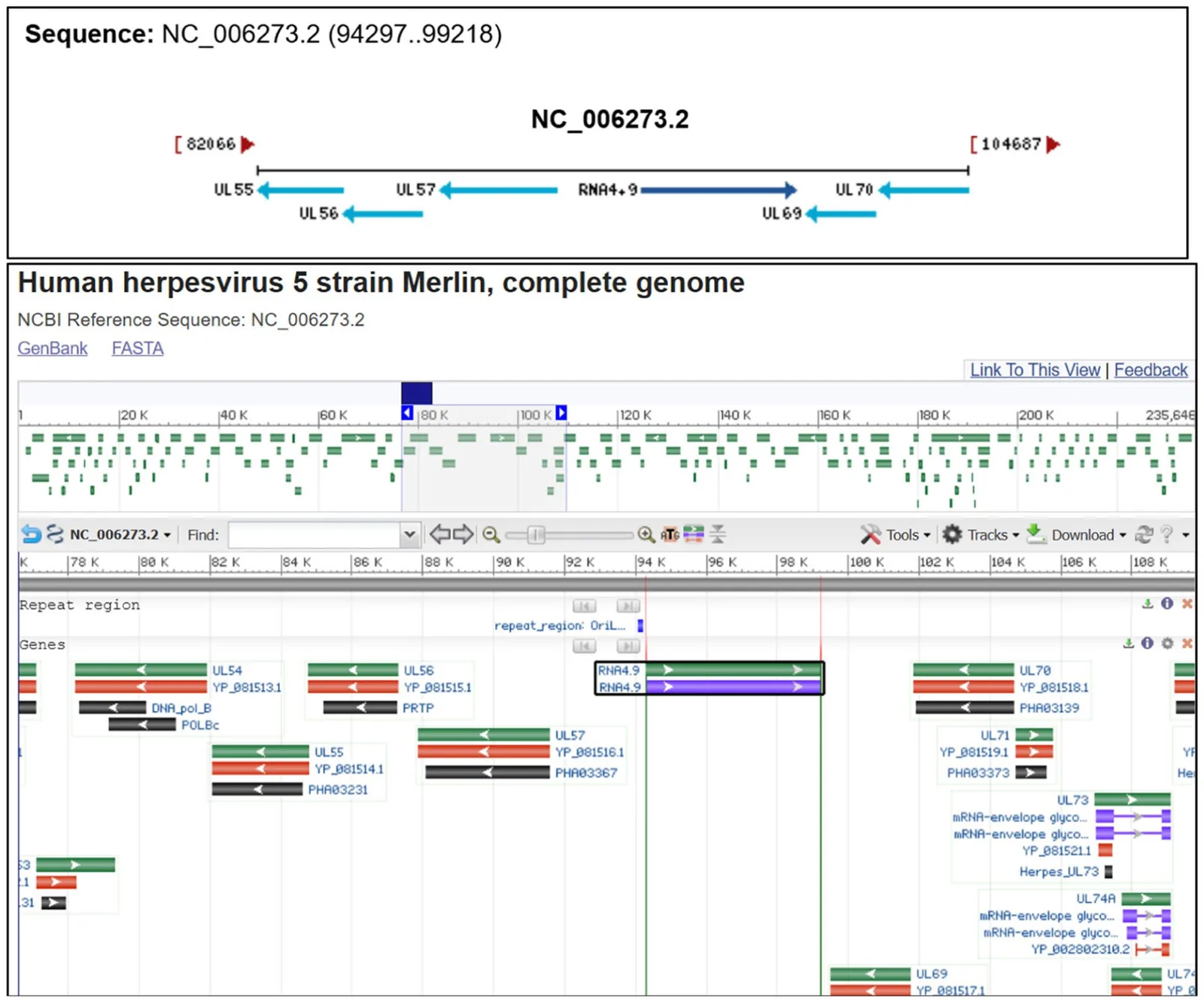
ACD RNAscope *lncRNA4.9* probe location. ACD RNAscope *lncRNA4.9* probe targeting the sequence of *lncRNA4.9* (*RNA4.9* pictured) of the human cytomegalovirus (Human betaherpesvirus 5) as referenced in NIH—National Library of Medicine: Gene https://www.ncbi.nlm.nih.gov/gene/13229470.

**Figure A2.**
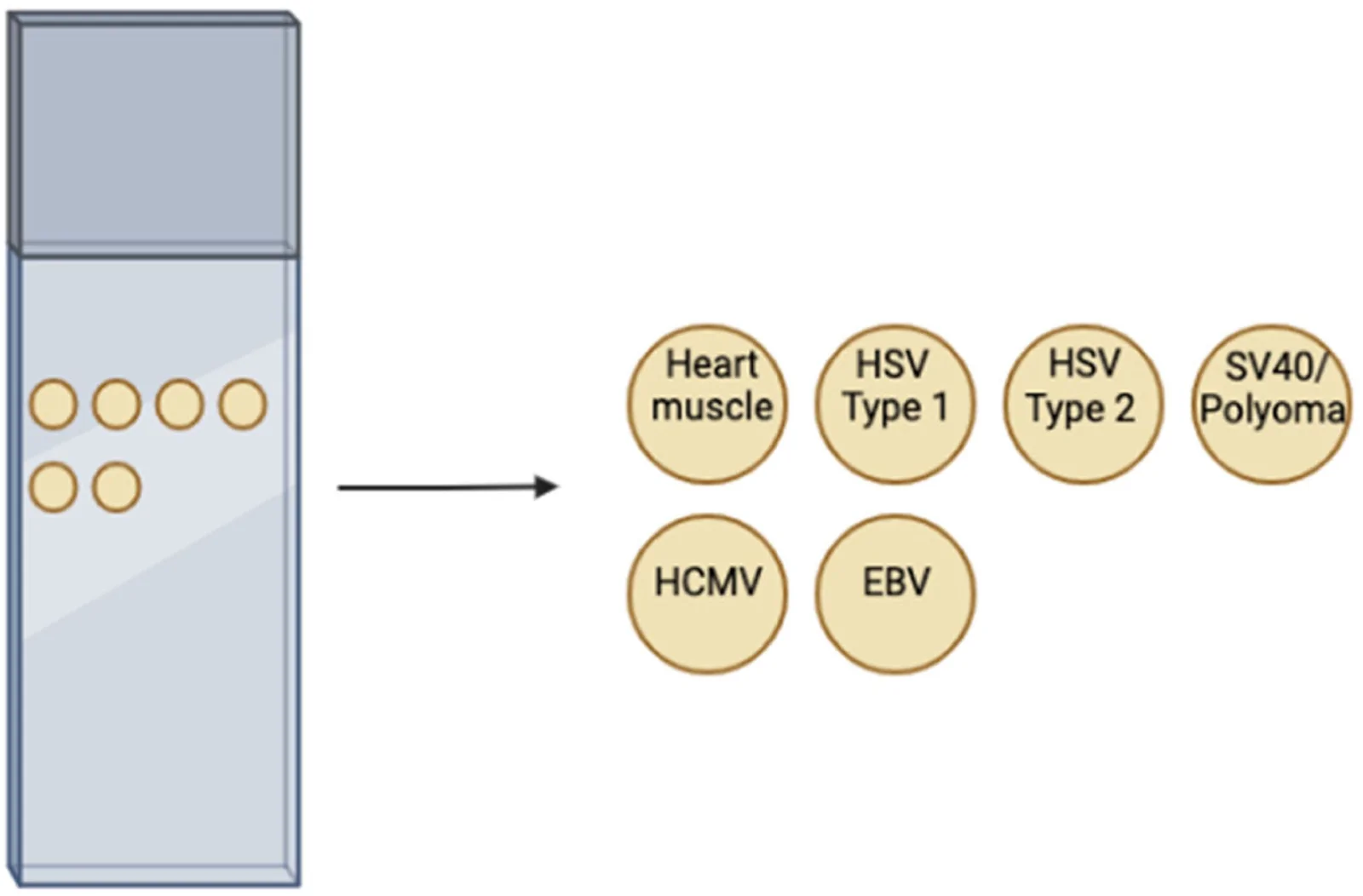
Schematic showing format of the control array. The control array comprised five cell line cores infected with a different virus: HSV type 1, HSV type 2, SV40/Polyoma, HCMV, and EBV. A heart muscle core was included (top left) to aid in orientation of the array. The formalin-fixed paraffin-embedded control array was cut into 5-µm-thick serial sections and mounted onto glass slides for use in the RNAscope 2.5 HD Assay (Figure created using Biorender.com).

**Figure A3.**
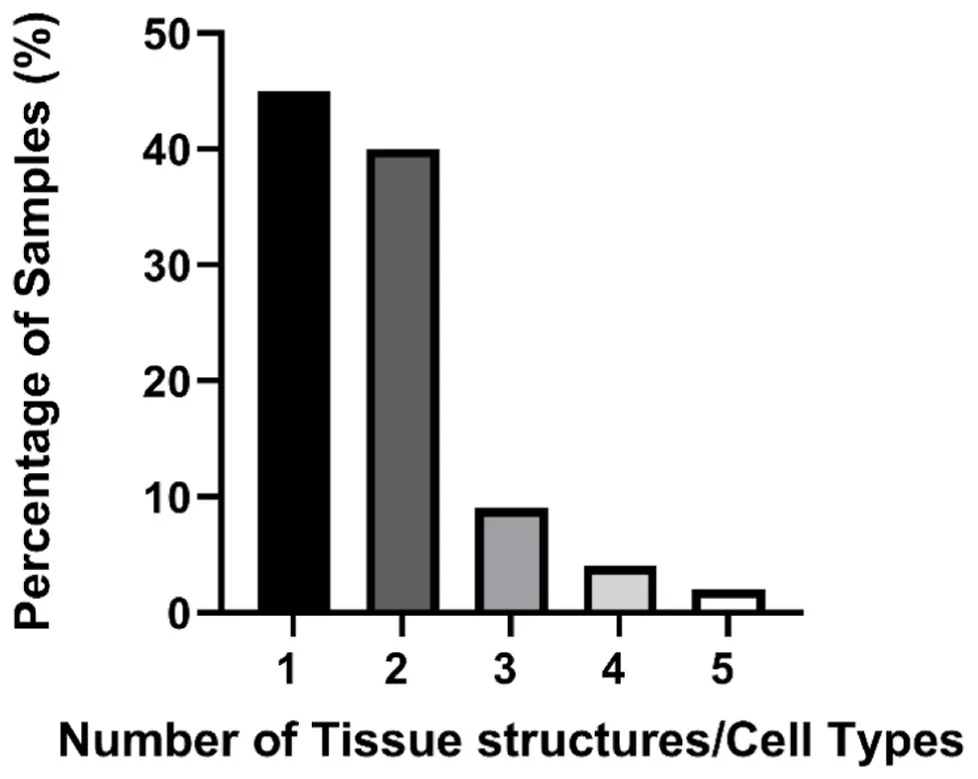
Distribution of positive *lncRNA4.9* hybridization signals among various tissue structures/cell types. Positive HCMV *lncRNA4.9* probe hybridization signals were detected in multiple tissue structures or cell types within each breast tumor sample. Detection in one breast tissue structure/cell type, *n*=21 (45%) tumor samples; two breast tissue structures/cell types, *n*=19 (40%) tumor samples; three breast tissue structures/cell types, *n*=4 (9%) tumor samples; four breast structures/cell types, *n*=2 (4%) tumor samples; five breast tissue structures/cell types, *n*=1 (2%) breast tumor sample.

**Figure A4.**
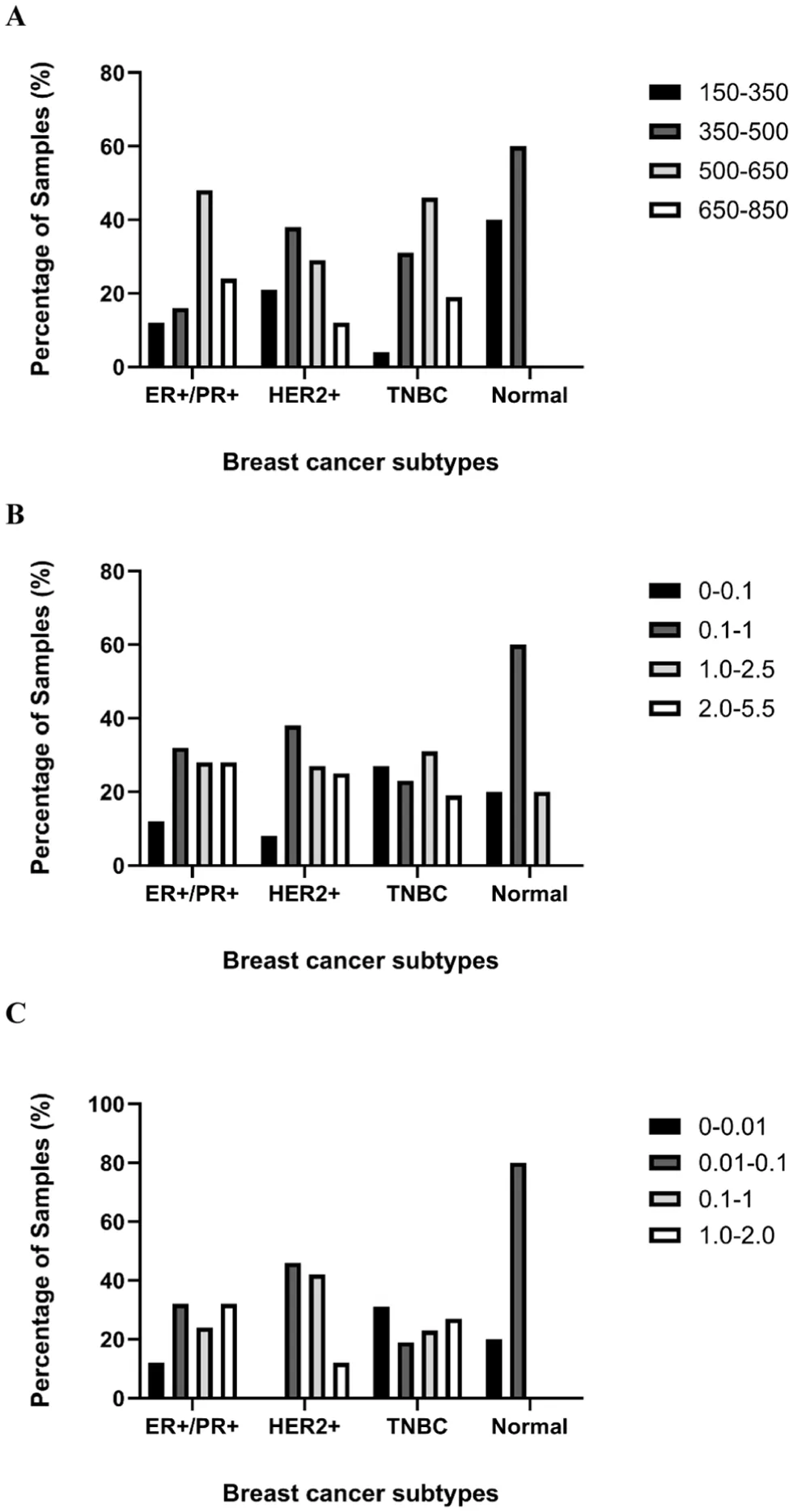
Number of cells per unit area and quantification of RNAscope HCMV *lncRNA4.9* probe hybridization signals. Breast tissue samples were analyzed using RNAscope assay to detect HCMV *lncRNA4.9* before analysis using the QuPath software package. Five representative areas were chosen per sample and analyzed for the number of probe hybridization signals per cell. The average value of the five areas for each sample was recorded and compared with the clinicopathological data and breast cancer clinical subtype of each patient sample. (A) The number of cells within samples of various clinical subtypes of breast cancer. (B) The number of RNAscope *lncRNA4.9* target probe hybridization signals within breast cancer clinical subtypes. (C) The number of RNAscope *lncRNA4.9* target probe hybridization signal clusters within breast cancer clinical subtypes (ER+/PR+, estrogen/progesterone receptor positive, *n*=25 samples; HER2+, HER2 overexpressing, *n*=24 samples; TNBC, triple-negative breast cancer, *n*=26 samples; Normal, normal breast tissue, *n*=5 samples).

**Figure A5.**
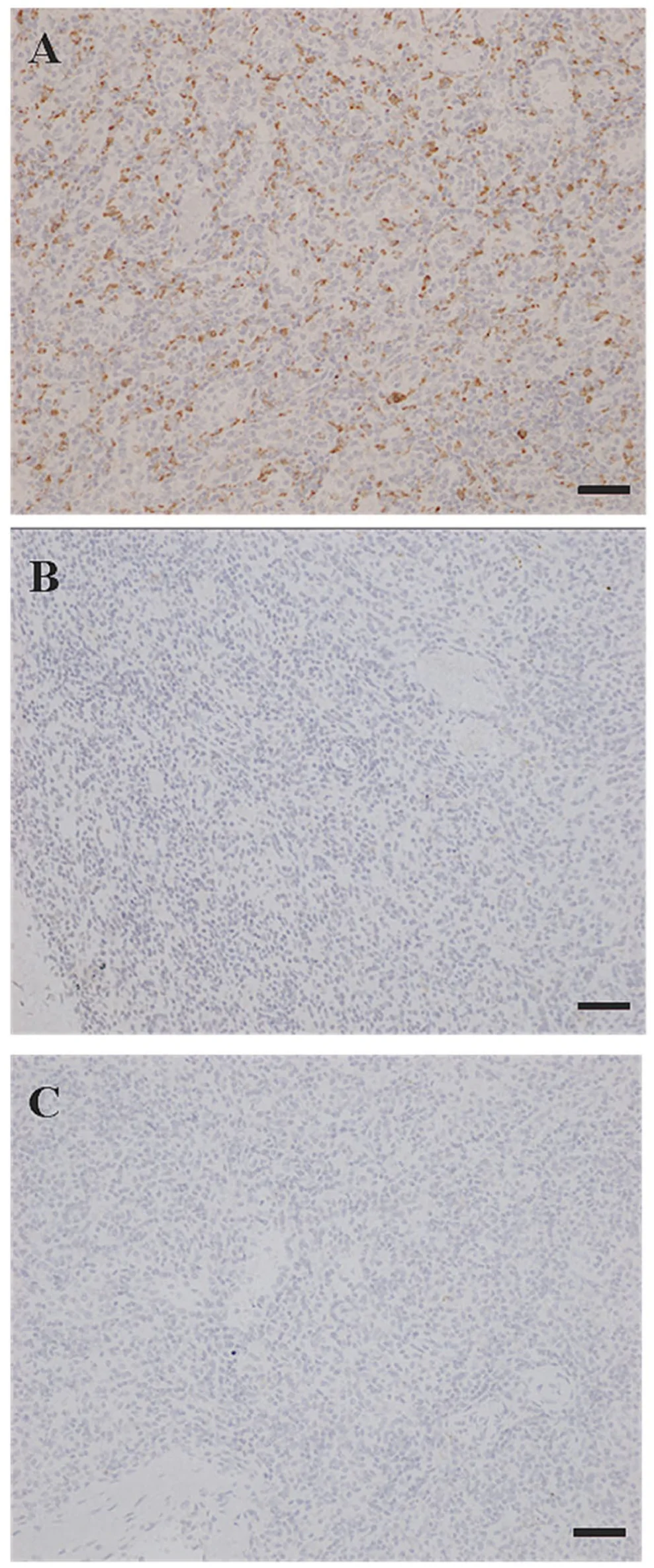
Positive and negative controls for anti-CD68 immunohistochemistry. Immunohistochemistry was conducted on serial sections of a control tissue array containing spleen, appendix, skin, and liver tissue. Representative images from spleen tissue show (A) anti-CD68 positive control (1:500 dil), (B) negative control treated with PBS only, and (C) mouse IgG Isotype negative control (1:500) (20× magnification; scale bar 50 µm).

## Appendix

**Table A1.** HCMV IgG Serology Status Within the Study Cohort, Subdivided According to Histological Subtype.

	ER+/PR+ *N* (%)	HER2+ *N* (%)	TNBC *N* (%)	Control *N* (%)
Positive	18/24 (75%)	9/22 (41%)	5/25 (20%)	1/5 (20%)
Negative	5/24 (21%)	13/22 (59%)	20/25 (80%)	4/5 (80%)
Equivocal	1/24 (4%)	0/22 (0%)	0/25 (0%)	0/5 (0%)
Not tested/serum unavailable	1	2	1	0
Total	25	24	26	5

**Table A2.** HCMV IgM Serology Status Within the Study Cohort, Subdivided According to Histological Subtype.

	ER+/PR+ *N* (%)	HER2+ *N* (%)	TNBC *N* (%)	Control *N* (%)
Positive	3/18 (17%)	10/19 (53%)	11/23 (48%)	2/4 (50%)
Negative	13/18 (72%)	6/19 (32%)	12/23 (52%)	2/4 (50%)
Equivocal	2/18 (11%)	3/19 (16%)	0/23 (0%)	0 (0%)
Not tested/serum unavailable	7	5	3	1
Total	25	24	26	5

**Table A3.** Detection of HCMV *lncRNA4.9* Probe in Various Tissue Types Within RNAscope Positive Breast Tumor Samples (*n*=47).

	Tissue Type Positive for HCMV *lncRNA4.9* RNAscope Probe; *n* (%)
Invasive tumor	27/47 (57%)
DCIS	13/47 (28%)
Breast ducts	16/47 (34%)
Adipose	15/47 (32%)
Stroma	14/47 (30%)
